# Molecular Mimicry of SecA and Signal Recognition Particle Binding to the Bacterial Ribosome

**DOI:** 10.1128/mBio.01317-19

**Published:** 2019-08-13

**Authors:** Lara Knüpffer, Clara Fehrenbach, Kärt Denks, Veronika Erichsen, Narcis-Adrian Petriman, Hans-Georg Koch

**Affiliations:** a Institute of Biochemistry and Molecular Biology, ZBMZ, Faculty of Medicine, Albert-Ludwigs-Universität Freiburg, Freiburg, Germany; b Faculty of Biology, Albert-Ludwigs-Universität Freiburg, Freiburg, Germany; Stony Brook University; Washington University School of Medicine

**Keywords:** SecA, SecY, protein transport, ribosomes, signal recognition particle

## Abstract

Bacteria execute a variety of protein transport systems for maintaining the proper composition of their different cellular compartments. The SecYEG translocon serves as primary transport channel and is engaged in transporting two different substrate types. Inner membrane proteins are cotranslationally inserted into the membrane after their targeting by the signal recognition particle (SRP). In contrast, secretory proteins are posttranslationally translocated by the ATPase SecA. Recent data indicate that SecA can also bind to ribosomes close to the tunnel exit. We have mapped the interaction of SecA with translating and nontranslating ribosomes and demonstrate that the N terminus and the helical linker domain of SecA bind to an acidic patch on the surface of the ribosomal protein uL23. Intriguingly, both also insert deeply into the ribosomal tunnel to contact the intratunnel loop of uL23, which serves as a nascent chain sensor. This binding pattern is remarkably similar to that of SRP and indicates an identical interaction mode of the two targeting factors with ribosomes. In the presence of a nascent chain, SecA retracts from the tunnel but maintains contact with the surface of uL23. Our data further demonstrate that ribosome and membrane binding of SecA are mutually exclusive, as both events depend on the N terminus of SecA. Our study highlights the enormous plasticity of bacterial protein transport systems and reveals that the discrimination between SRP and SecA substrates is already initiated at the ribosome.

## Abstract (precis)

Bacterial protein transport via the conserved SecYEG translocon is generally classified as either cotranslational, i.e., when transport is coupled to translation, or posttranslational, when translation and transport are separated. We show here that the ATPase SecA, which is considered to bind its substrates posttranslationally, already scans the ribosomal tunnel for potential substrates. In the presence of a nascent chain, SecA retracts from the tunnel but maintains contact with the ribosomal surface. This is remarkably similar to the ribosome-binding mode of the signal recognition particle, which mediates cotranslational transport. Our data reveal a striking plasticity of protein transport pathways, which likely enable bacteria to efficiently recognize and transport a large number of highly different substrates within their short generation time.

## INTRODUCTION

Protein targeting to the universally conserved SecYEG translocon in bacteria is generally considered to occur either cotranslationally by the signal recognition particle (SRP), a ribonucleoprotein complex consisting in Escherichia coli of the protein Ffh and the 4.5S RNA, or posttranslationally by the ATPase SecA (1–4). While SRP targets predominantly aggregation-prone inner membrane proteins, SecA is responsible for the targeting of less hydrophobic periplasmic and outer membrane proteins, collectively called secretory proteins (5). Cotranslational protein transport is limited by the low translation rate (6), and as a consequence, a large portion of the (anyway) small number of SecYEG translocons (1) are engaged by translating ribosomes. The execution of a posttranslational transport pathway probably enables cells to rapidly translocate proteins whenever a SecYEG translocon is available.

Central to the cotranslational targeting strategy is the ability of SRP to bind to ribosomes and to scan translating ribosomes for the presence of a hydrophobic signal sequence indicating that a membrane protein is emerging (7, 8). Binding of SRP to the ribosome primarily involves the ribosomal protein uL23 (9), which is located at the ribosomal tunnel exit. One particular feature of the bacterial uL23 homologue is that it contains a hairpin-like loop that extends into the ribosomal tunnel, approximately 20 Å away from the tunnel exit. Recent data demonstrate that the C terminus of Ffh inserts into the ribosomal tunnel to contact this hairpin loop (10). This contact allows SRP to scan ribosomes very early for potential substrates, even before the signal anchor sequence is fully exposed to the outside of the ribosome (11). Once SRP has recognized its substrate, the ribosome-nascent chain (RNC) complex is targeted to the SecY-bound SRP receptor FtsY (12, 13), and the RNC is handed over to SecYEG for insertion (14). Lipid insertion of hydrophobic transmembrane domains (TMs) is a thermodynamically favored reaction (15) that is further supported by the translational activity of the ribosome and by YidC, which is located at the lateral gate of SecY and aids lipid insertion of TMs (16–18). For the translocation of large periplasmic loops in membrane proteins, additional energy is provided by the ATPase activity of SecA (19–22), although the exact timing of SecA binding to hydrophilic loops in membrane proteins and its coordination with ongoing translation by the SecY-docked ribosome are still unknown.

Despite its role in translocating periplasmic domains of membrane proteins, SecA has been primarily associated with the posttranslational targeting and translocation of secretory proteins (2). Based on the current model, secretory proteins are shielded during their synthesis by the ribosome-bound chaperone trigger factor (TF) (23–25), which binds, like SRP, to uL23 (26). Recent data indicate that optimal binding of TF occurs once the nascent chain reaches a length of approximately 100 amino acids (27). After their release from the ribosome, secretory proteins are then captured by the SecYEG-bound SecA (28–30), which translocates the secretory protein in ATP-dependent steps across the SecYEG channel (31–33). Some secretory proteins require the export-specific chaperone SecB for keeping them in transport-competent conformation during targeting to SecA (34), and SecB can interact with its substrates before they are released from the ribosome (34).

Different from the SRP pathway, the transport of secretory proteins occurs even when it is uncoupled from protein synthesis (35, 36). Furthermore, efficient targeting and transport require the exposure of mature parts of the substrate (37), which supports a posttranslational translocation mode by the SecA/SecY pathway. However, the generally accepted posttranslational transport of secretory proteins by SecA does not exclude a cotranslational interaction of SecA with its substrates. Indeed, cotranslational contacts between SecA and nascent secretory proteins were shown both *in vitro* (25, 38) and *in vivo* (39). This is further supported by data showing that SecA can bind to ribosomes and that this interaction enhances the efficient translocation of the secretory maltose-binding protein (MBP) (40, 41). Cotranslational binding of SecA does not seem to be limited to secretory proteins because SecA also mediates the cotranslational transport of the membrane protein RodZ (42, 43). This confirms earlier data showing that the role of SecA during membrane insertion of single-spanning membrane proteins is not restricted to the translocation of periplasmic loops (44).

The observation that both SRP and SecA bind to the ribosome for cotranslationally recognizing their substrates imposes important questions about how substrates are selected and processed at the tunnel exit.

## RESULTS

### SecA makes multiple contacts with the ribosomal protein uL23 and enters into the ribosomal peptide tunnel.

**FIG 1:**
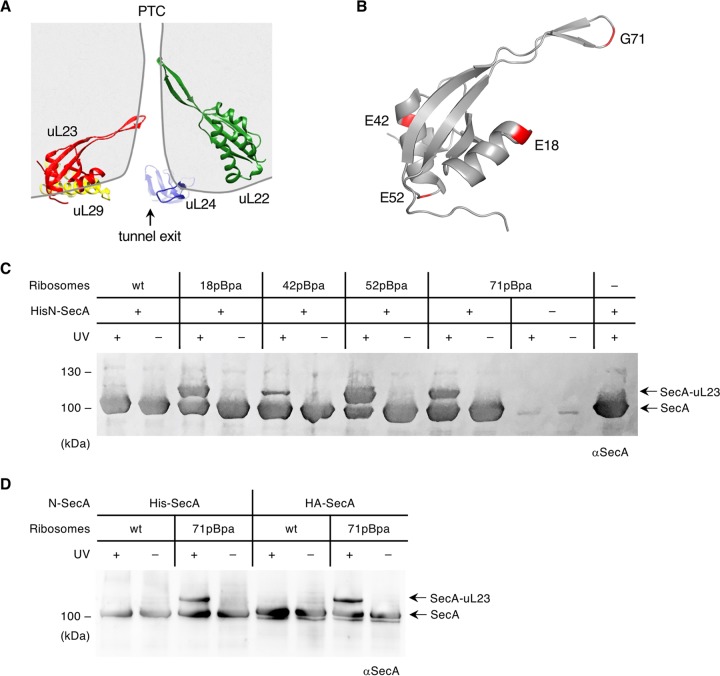
Cross-linking of SecA and uL23. (A) Cartoon showing the polypeptide exit tunnel in the 50S subunit of the bacterial ribosome. The nascent protein passes the loops of ribosomal proteins uL4 (not shown) and uL22 (green) on its path through the tunnel and shortly before emerging from the ribosome also the loop of uL23 (red). The tunnel exit area is surrounded by the proteins uL23, uL29 (yellow), and uL24 (blue). PTC, peptidyltransferase center. The 50S model is derived from PDB ID 4YBB (93). (B) Residues of E. coli uL23 that were replaced with pBpa are shown in red. The uL23 template is derived from PDB ID 4V6M (72). (C) Purified SecA and ribosomes (both 500 nM) were combined, and half of each sample was exposed to UV light for cross-link induction (+UV), whereas the other half was kept in the dark (−UV). Wild-type (wt) ribosomes and the ribosomes bearing the cross-linker pBpa at different positions of uL23 are indicated. Samples were separated by SDS-PAGE and detected with immunoblotting using anti-SecA antibodies (αSecA). SecA and the cross-links with uL23 (SecA-uL23) are designated with arrows. (D) The N-terminal His tag of SecA was replaced with the N-terminal hemagglutinin tag (HA-SecA), and the cross-linking experiment was repeated as in panel B. Representative blots of at least three independent replicates are shown.

For analyzing the SecA-ribosome interaction in detail, we focused on the ribosomal protein uL23, which is located at the ribosomal tunnel exit (Fig. 1A) and which was previously shown to be a hot spot for binding of targeting factors, chaperones, and processing enzymes (45, 46). Ribosomes containing the UV-dependent photo-cross-linker *para*-benzoyl-l-phenylalanine (pBpa) incorporated at different positions within uL23 (Fig. 1B) were generated *in vivo* and purified (10, 47, 48). These ribosomes were incubated with purified SecA, exposed to UV light, and subsequently analyzed by immunodetection using anti-SecA antibodies. UV-dependent cross-linking products of approximately 115 kDa were observed with ribosomes containing pBpa at the surface-exposed positions E18, E42, and E52 but also for residue G71 at the tip of the intratunnel loop of uL23 (Fig. 1C). No cross-links were observed when SecA was incubated with wild-type ribosomes lacking pBpa or when pBpa containing ribosomes were exposed to UV light in the absence of SecA. UV exposure of just SecA did also not show cross-linking products (Fig. 1C).

SecA used in these experiments contained an N-terminal His tag, and protonation of the imidazole nitrogen atoms could favor the interaction with the negatively charged rRNA on the ribosomal surface or within the ribosomal tunnel. This was excluded by repeating the cross-link experiment with a SecA derivative that contained an N-terminal hemagglutinin (HA) tag instead of the His tag. With both SecA variants, the UV-dependent cross-link to the intratunnel residue G71 was observed (Fig. 1D). In summary, these data demonstrate that SecA not only contacts the surface of uL23 but also penetrates deeply into the ribosomal tunnel, a feature that has been observed before for SRP but not for other uL23-interacting proteins like trigger factor, peptide deformylase (PDF), or methionine aminopeptidase (MAP) (10, 49).

### Identification of the ribosome-binding site in SecA.

**FIG 2:**
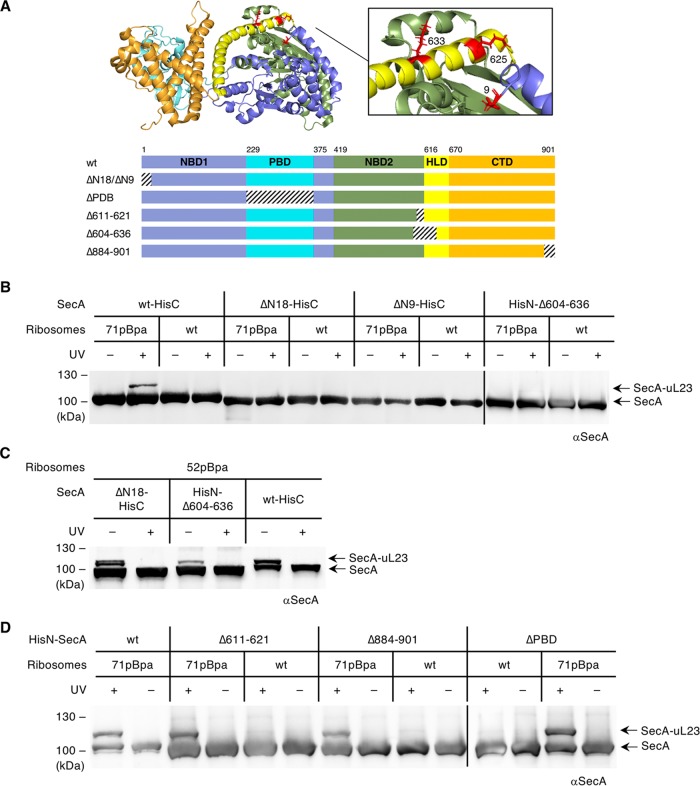
Identification of the ribosome-binding site of SecA. (A) Structure of SecA from E. coli (PDB ID 2VDA [56]), consisting of nucleotide-binding domains 1 and 2 (NBD 1 and NBD 2, respectively), the peptide binding domain (PBD), the helical linker domain (HLD), and the C-terminal domain (CTD). The N terminus is not completely resolved in the E. coli structure; therefore, residue 9 is colored in red instead of residue 5 that was replaced with pBpa. Residues 625 and 633, which were implicated in uL23 binding, are also shown in red. The bottom shows the schematic domain organization of wild-type (wt) SecA (40) and the deletion variants. The deleted parts are indicated by dashed lines. (B) uL23(71pBpa)-bearing 70S ribosomes were incubated at equimolar concentrations (500 nM) with purified wt SecA or SecA deletion variants. ΔN9 and ΔN18 refer to N-terminal deletions of 9 and 18 amino acids, respectively. Wild-type SecA and both mutants carry a C-terminal His tag. Δ604–636 refers to a deletion which includes residues K^625^ and K^633^ and contains the His tag at the N terminus. Cross-linking and the subsequent analyses were performed as described in Fig. 1. SecA and the cross-links to uL23 (SecA-uL23) are designated with arrows. (C) Cross-linking as in panel B but with pBpa incorporated in the surface-exposed residue 52 of uL23. (D) Cross-linking as in panel B between uL23(71pBpa) ribosomes and SecA variants lacking either amino acids 611 to 621, the C terminus (Δ884–901), or the PBD. Representative blots of at least two independent replicates are shown.

Previous attempts to identify the ribosome-binding site of SecA led to conflicting results. Based on biochemical analyses using different SecA mutants, it was proposed that the helical linker domain (HLD) (Fig. 2A) and, in particular, residues K625 and K633, are required for ribosome-uL23 binding (40). In contrast, cryo-electron microscopy (cryo-EM) reconstructions of SecA bound to the ribosome indicated that SecA interacts via its N terminus with uL23 (50). Although an interaction with the ribosomal tunnel interior is more easily envisioned for either the N terminus or the C terminus of SecA, the distal part of the tunnel is wide enough to accommodate two α-helices in a hairpin-like structure (51, 52). To identify the ribosome-binding site within SecA and, in particular, the residues that insert into the ribosomal tunnel, several deletion and truncation mutants of SecA were generated (Fig. 2A).

The N terminus of SecA has been shown to be important for the interaction with the SecYEG translocon and phospholipids (53–55). However, the first 8 amino acids were not resolved in the crystal structure of E. coli SecA (56), suggesting a certain flexibility that could favor the interaction with the intratunnel loop. Therefore, two N-terminal deletion mutants were generated which lacked either the first 9 amino acids (ΔN9) or even a longer stretch of 18 amino acids (ΔN18). These SecA variants contained a C-terminal His tag and were compared with a C-terminally His-tagged wild-type SecA. The cross-link to position 71 of uL23 was observed for wild-type SecA, demonstrating that the position of the His tag does not influence cross-linking to uL23. However, no cross-link was detected with the two SecA N-terminal deletions (Fig. 2B). This would support the hypothesis that it is the N terminus of SecA that protrudes into the ribosomal tunnel. However, when parts of nucleotide-binding domain 2 (NBD2) and the HLD (residues 604 to 636) were deleted, which includes the two lysine residues that were proposed to be involved in ribosome binding (Fig. 2B), there was also no detectable cross-link to the intratunnel residue G71.

To exclude the possibility that the deletions interfered with the general ability of SecA to bind to ribosomes, the cross-link experiment was repeated with ribosomes carrying pBpa at position 52 located on the ribosomal surface. For the SecA ΔN18 and Δ604–636 variants, we observed cross-linking products (Fig. 2C), indicating that the ability to bind to the ribosome is retained when the N terminus or parts of the NBD2 and HLD are deleted. So far, these data did not allow for a clear identification of the SecA domain that inserts into the ribosomal tunnel, and consequently, three additional SecA truncations were tested. In SecA(Δ611–621), only smaller parts of the NBD2 and the HLD were deleted. These residues form a loop close to the predicted ribosome-interacting residues K625 and K633 (40), and this loop would in principle be flexible enough to enter the ribosomal tunnel. In contrast to the SecA(Δ604–636) variant, this variant was still able to contact the intratunnel loop of uL23 (Fig. 2D). For two other SecA variants that either lacked the C terminus (residues 884 to 901) or the peptide-binding domain (PBD; Δ233–365) (57), the intratunnel contact was also not impaired (Fig. 2D).

**FIG 3:**
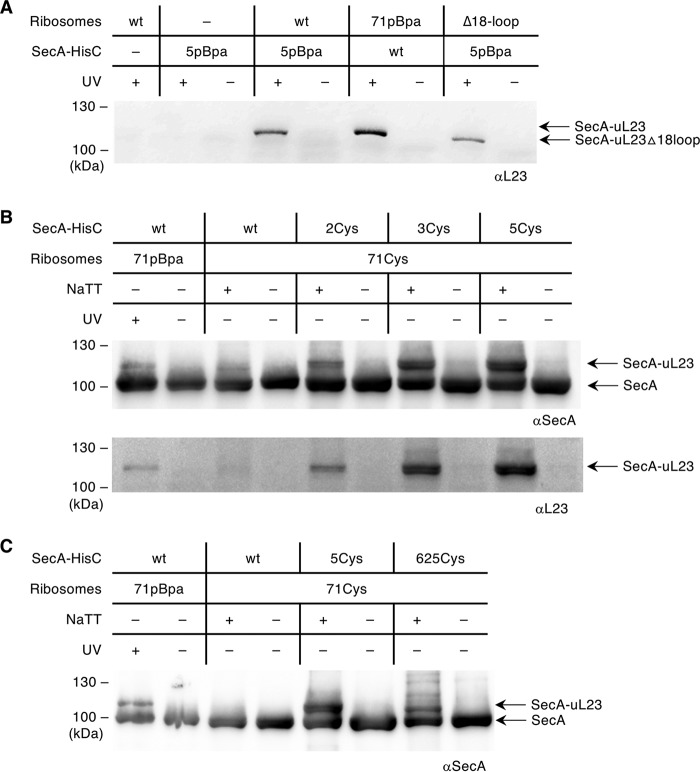
The N terminus of SecA and the helical linker domain contact the intratunnel loop of uL23. (A) Purified SecA bearing pBpa at position 5 at the N terminus (5pBpa) was incubated with wild-type ribosomes (wt) or ribosomes lacking 18 amino acids of the intratunnel loop of uL23 (Δ18-loop). Samples were exposed to UV light and analyzed as described in Fig. 1. Wild-type ribosomes or SecA(5pBpa) were exposed to UV light and served as controls. As an additional control, wild-type SecA was incubated with ribosomes bearing pBpa at position 71 of uL23 (71pBpa) and cross-linked. The cross-linking products were detected with anti-L23 antibodies after SDS-PAGE and Western blotting. (B) The contact of the N terminus of SecA with the inside of the ribosomal tunnel was verified by cysteine cross-linking. Ribosomes containing cysteine in position 71 of uL23 (71Cys) were incubated with purified SecA mutants bearing a cysteine residue at the N terminus at position 2, 3, or 5 but that lacked all three native cysteine residues at the C terminus (SecA_His_C^885^S-C^887^S-C^896^S). Disulfide bridge formation was induced with sodium tetrathionate (NaTT). The control samples (−) were kept reduced with TCEP [Tris(2-carboxyethyl)phosphine]. Samples were separated under nonreducing conditions, blotted, and decorated with anti-SecA and anti-L23 antibodies. (C) Disulfide cross-linking was repeated with a SecA variant that had cysteine incorporated at position 625 within the helical linker domain. The experiment was performed as described in panel B. Representative blots of at least three independent replicates are shown.

To determine whether the N terminus of SecA inserts into the ribosomal tunnel, the cross-linking strategy was reversed, and pBpa was inserted into the N-terminal amino acid at position 5 of SecA. SecA(5pBpa) was purified, incubated with wild-type ribosomes, and exposed to UV light. Immunodetection with anti-uL23 antibodies revealed a UV-dependent cross-linking product of approximately 115 kDa for SecA(5pBpa) that was not observed in the absence of ribosomes. Importantly, the migration of this cross-link product on SDS-PAGE gels was identical to the product observed with wild-type SecA and uL23(71pBpa)-containing ribosomes (Fig. 3A). When ribosomes lacking the intratunnel loop of uL23 (Δ18-loop) were incubated with SecA(5pBpa), the cross-link product was still observed, although it migrated slightly faster due to the absence of the loop (Fig. 3A). This indicates that the N terminus of SecA is still able to contact uL23 when the intratunnel loop is missing, which is in line with our observation that SecA not only contacts the intratunnel loop but also the surface-exposed parts of uL23 (Fig. 2C).

The hypothesis that the N terminus of SecA protrudes into the ribosomal tunnel was directly verified by using cysteine cross-linking. To this end, uL23 variants with a cysteine residue at position 71 were generated and incubated with SecA that contained engineered cysteine residues at three different N-terminal positions, and disulfide bond formation was induced by adding the oxidant sodium tetrathionate (NaTT). For all three SecA variants, a NaTT-dependent band of 115 kDa was observed on nonreducing gels. This band was identical to the UV-dependent band observed with uL23(71pBpa) and not observed with a SecA variant that only contained the endogenous cysteine at position 98 (Fig. 3B).

Using the same approach with a SecA variant that contained an engineered cysteine residue at position 625 also showed a NaTT-dependent cross-link product (Fig. 3C), demonstrating that the N terminus of SecA is in contact with the uL23 intratunnel loop but also with residues of the HLD. These data consolidate conflicting observations (40, 50) about the domains of SecA that interact with the ribosomal peptide tunnel exit. Our data demonstrate that both the N terminus of SecA and parts of the HLD can insert about 20 Å into the ribosomal tunnel where they contact the intratunnel loop of uL23. The N terminus of SecA and the HLD are located in close vicinity to each other (Fig. 2A), and thus, the SecA structure supports our observation that both can protrude into the ribosomal tunnel. Furthermore, protrusion of either the N terminus or the HLD into the ribosomal tunnel is likely favored by the intrinsic conformational dynamics of SecA (58–60). Whether this occurs simultaneously or sequentially requires further analyses. Nevertheless, the protrusion of SecA into the ribosomal peptide tunnel is surprisingly similar to the recently observed scanning of the ribosomal tunnel by SRP, which involves the deep penetration of SRP’s C terminus into the tunnel (10).

### SRP and SecA compete for ribosome binding.

**FIG 4:**
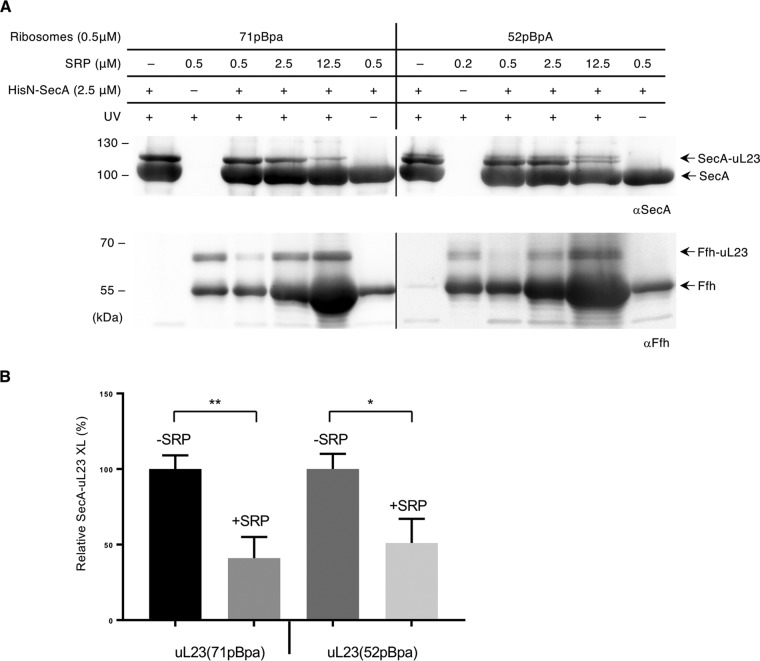
SecA and Ffh compete for binding to uL23 in vacant ribosomes. (A) Purified SecA and Ffh were premixed in different ratios as indicated and incubated with ribosomes bearing pBpa at position 71 or 52. Subsequently, UV exposure was performed as described in Fig. 1. The cross-linking products were detected with anti-SecA (top blots) and anti-Ffh (bottom blots) antibodies after SDS-PAGE and are labeled with arrows. Representative blots of at least three independent replicates are shown. (B) Quantification of cross-linking efficiency between SecA and uL23 in the presence and absence of SRP. The amount of the SecA-uL23 cross-linking product for either position 71 or position 52 was quantified after immunodetection using the ImageQuantTL/ImageJ software, and the amount of cross-linked material in the presence of 0.5 μM ribosomes and 2.5 μM SecA was set to 100%. The amount of SecA-uL23 cross-linked material in the presence of 2.5 μM SRP was then calculated. The values correspond to the mean of at least three independent replicates, and the standard deviation is indicated by error bars. *P* values were calculated with an unpaired *t* test (*n* ≥ 3). **, *P* < 0.005; *, *P* < 0.05.

To determine whether SRP and SecA compete for access to the ribosomal tunnel in nontranslating ribosomes, a defined concentration of SecA (2.5 μM) was premixed with SRP at increasing concentrations and then incubated with pBpa-containing ribosomes (0.5 μM). Cross-linking was induced by UV exposure, and cross-links to either SecA or Ffh, the protein component of the bacterial SRP, were visualized by immunodetection. Increasing the SRP concentration reduced the amount of the SecA-uL23 cross-linking product for both position 71 within the ribosomal tunnel and position 52 at the ribosomal surface (Fig. 4A). Simultaneously, the uL23-Ffh cross-linking product increased. Quantification of the SecA-uL23 cross-linking efficiency revealed an approximately 50% reduction when SecA and SRP were present at equimolar concentrations (Fig. 4B).

Thus, SRP and SecA compete for binding to nontranslating ribosomes, which is in line with the observation that they use similar binding sites on the ribosomes.

### SecA retracts from the ribosomal tunnel in translating ribosomes.

**FIG 5:**
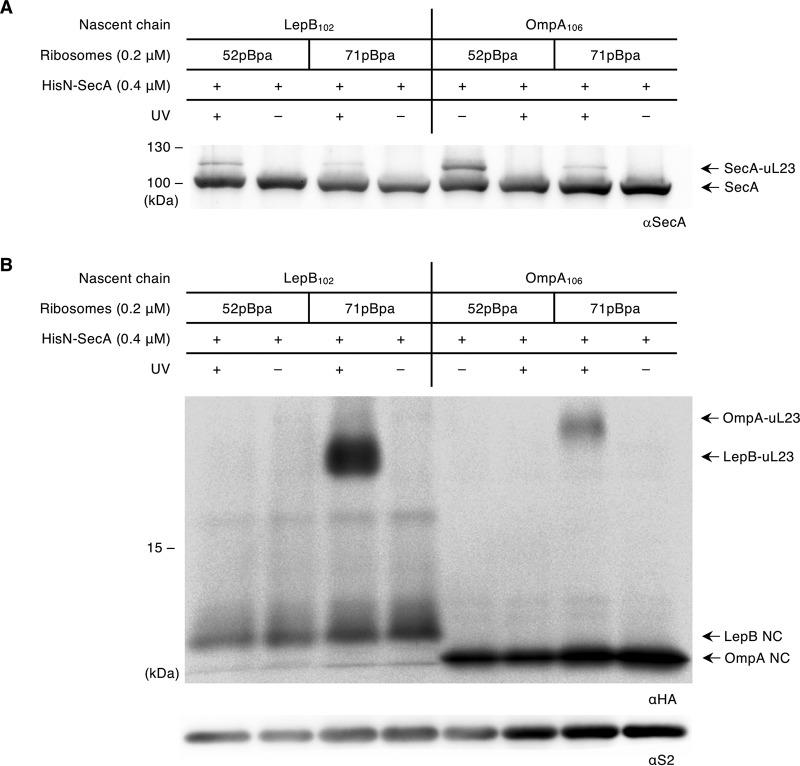
SecA retracts from the tunnel interior in the presence of a nascent chain but maintains contact with the ribosomal surface. (A) LepB- and OmpA-ribosome-nascent chains (RNCs; 0.2 μM) bearing the cross-linker pBpa at position 71 or 52 of uL23, as indicated, were incubated with SecA (0.4 μM). Cross-linking was induced by UV light; the material was separated by SDS-PAGE and analyzed by immunoblotting. Anti-SecA antibodies detect the cross-linking product of SecA and uL23, as indicated. (B) The material shown in panel A was decorated with anti-HA antibodies for detection of the nascent chains via their N-terminal HA tag and the cross-links between uL23 and the RNCs. The ribosomal protein S2 (anti-S2 antibodies) served as a loading control. One representative figure of at least three replicates is shown.

The discovery of SecA contacting the intratunnel loop in nontranslating ribosomes raised the question on whether this contact would be maintained in translating ribosomes. This was analyzed by using ribosome-nascent chains (RNCs) in which pBpa was inserted either within the tunnel (position 71) or at the surface (position 52) of uL23. The classical SecA-dependent outer membrane protein OmpA and the SecA-dependent inner membrane protein LepB were selected as the substrates. Although both substrates are SecA dependent, OmpA requires SecA for both targeting and translocation, while LepB is targeted by SRP, and SecA is required for the subsequent translocation of the large C-terminal periplasmic domain. For both RNCs, SecA cross-links to the surface-exposed residue 52 of uL23 were observed, but no significant cross-links to the inner tunnel residue 71 were found (Fig. 5A). Instead, this residue cross-linked to the nascent chain of LepB and OmpA, respectively (Fig. 5B). These data indicate that once the ribosomal tunnel is filled with a nascent chain, SecA loses contact with the intratunnel loop but stays in contact with the surface of uL23. This retraction seems to be independent of the nature of the approaching nascent chain, because it is observed for both OmpA and LepB. Thus, as previously observed for SRP (10), the insertion of SecA into the ribosomal tunnel does not enable SecA to decode the sequence information of the nascent chain.

### Membrane-bound SecA is unable to bind to ribosomes.

**FIG 6:**
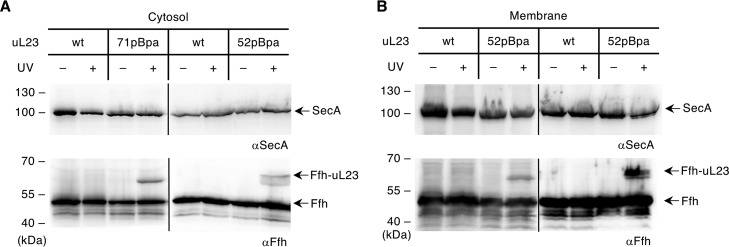
*In vivo*, the majority of SecA is not in contact with the ribosome. (A) *In vivo* cross-linking of E. coli cells expressing either wild-type uL23 or uL23 variants containing pBpa either at position 52 or 71. Exponentially growing cells were harvested, and one-half of each culture was exposed to UV light, while the other was kept in the dark. After cell breakage, the cytosolic fraction was centrifuged in high-salt buffer through a 50% sucrose cushion for ribosome enrichment. After SDS-PAGE and Western blotting, the membrane was decorated with anti-SecA (top blots) and anti-Ffh (bottom blots) antibodies. (B) As in panel A, but after cell breakage, the membrane fraction was enriched via sucrose gradient centrifugation and analyzed as in panel A. Representative blots of at least three independent replicates are shown.

The data demonstrate that SRP and SecA execute an identical binding mode to translating and nontranslating ribosomes and support cotranslational substrate recognition by both targeting factors. To address to what extent SecA and SRP interact with ribosomes in living E. coli cells, the cross-linking approach was performed *in vivo*. uL23(71pBpa) was expressed in E. coli strain MC4100 Δ*rplW*, which lacks the chromosomal uL23 gene but contains either plasmid-carried wild-type *rplW* or a *rplW* variant with a TAG stop codon for pBpa insertion. During growth, pBpa was added to the medium, and after UV exposure of whole cells, cytosolic ribosomes were isolated and analyzed for cross-links to either SecA or SRP. While cross-links to SRP were easily detectable, no cross-link to SecA was visible (Fig. 6A). Repeating the approach with uL23(52pBpa)-expressing cells showed the same results, that *in vivo* cross-links were detected to SRP but not to SecA. When the membrane fraction of these cells was analyzed, cross-links to SRP were observed for both uL23(52pBpa) and uL23(71pBpa), but no cross-link to SecA was observed (Fig. 6b). This indicates that the majority of SRP is in contact with either cytosolic or membrane-bound ribosomes *in vivo*, while under these conditions, the fraction of SecA that is in stable contact with ribosomes is too low to be detected by the cross-linking approach. It is important to emphasize that SecA is only peripherally attached to the membrane and partly released during cell breakage. Therefore, the SecA content in cytosolic extracts (Fig. 6A) represents the sum of the truly soluble SecA fraction and the SecA fraction that is released from the membrane during cell breakage.

**FIG 7:**
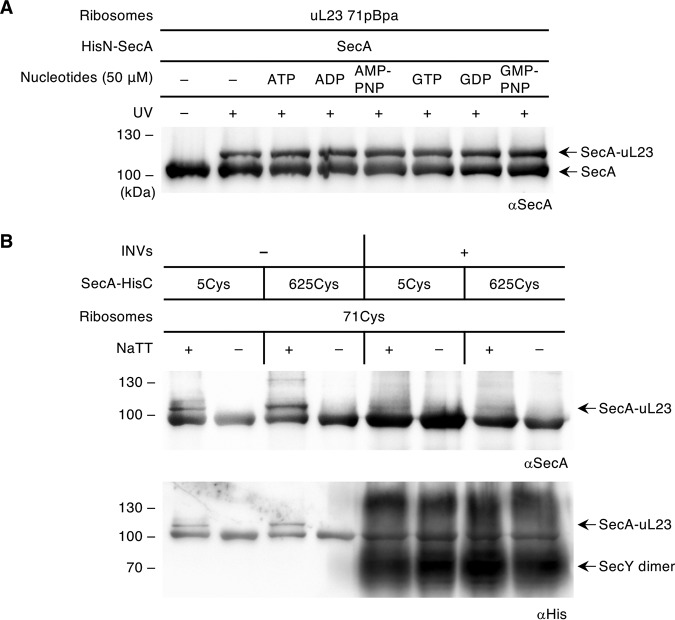
The SecA-ribosome contact is nucleotide independent and dissociated by the presence of membranes. (A) Purified SecA (500 nM) and equimolar amounts of ribosomes bearing pBpa at position 71 of uL23 were cross-linked as described in Fig. 1 in the presence of different nucleotides (50 μM), as indicated. The cross-linking products were analyzed by SDS-PAGE and decorated with anti-SecA antibodies. One representative figure of two replicates is shown. (B) *In vitro* cysteine cross-linking was performed as described in Fig. 3B with SecA mutants (1 μM) containing a cysteine residue at either position 5 or 625 to ribosomes with a cysteine residue at position 71 of uL23. When indicated, SecA was preincubated with inner membrane vesicles (INVs) generated from a SecYEG-YidC-overexpressing strain. The final SecYEG concentration was 2 μM. After incubation, unbound SecA was removed by centrifugation, ribosomes were added, and cross-linking was induced by UV exposure. Cross-links were separated by SDS-PAGE and analyzed by immunoblotting. The SecA variants and the overexpressed SecY in the INVs carried a His tag and were detected with anti-His antibodies. One representative figure of at least three replicates is shown.

SecA is an ATPase that is preferentially localized to the bacterial membrane due to its affinity to anionic phospholipids and to the SecYEG translocon (61, 62). Thus, the inability to detect uL23 cross-links to SecA *in vivo* could reflect a membrane- or nucleotide-induced SecA conformation that prevents ribosome binding. This was analyzed *in vitro* by incubating purified SecA with uL23(71pBpa) ribosomes in the presence of different nucleotides and subsequent UV exposure. The 115-kDa SecA-uL23 cross-link was detected in the absence of nucleotides, as seen before (Fig. 7A), but also in the presence of ATP, ADP, or the nonhydrolyzable ATP analogue adenylyl-imidodiphosphate (AMP-PNP) (Fig. 7A). The addition of GTP, GDP, or guanosine 5′-[β,γ-imido]triphosphate (GMP-PNP) also did not influence the formation of the cross-link product. Thus, although nucleotides have been shown to influence the conformation of SecA (58, 59), the contact of SecA with the ribosomal tunnel appears to be nucleotide independent.

Whether membranes influence the SecA-ribosome interaction was analyzed by testing cysteine cross-linking between SecA and uL23(71Cys) ribosomes in the presence and absence of inner membrane vesicles (INVs). In the absence of INVs, the cross-link to the intratunnel loop was detectable for both SecA(5Cys) and SecA(625Cys), as seen before. However, when SecA was preincubated with INVs, followed by centrifugation for removing unbound SecA, no SecA cross-link to uL23 was observed (Fig. 7B). This indicates that only soluble SecA binds to ribosomes, while the membrane/SecYEG-bound SecA exists in a conformation that prevents binding to the intratunnel loop.

Considering that the N terminus of SecA is involved in the interaction with phospholipids, SecY, and the chaperone SecB (63–65), our observation that the N terminus also binds to ribosomes suggests that the N terminus acts as a major switch that regulates the multiple steps of protein translocation across the bacterial membrane.

## DISCUSSION

Protein targeting in bacteria is generally viewed as a dichotomous process in which secretory proteins posttranslationally engage the ATPase SecA, while inner membrane proteins are cotranslationally targeted by the GTPase SRP (1, 66). The two pathways converge at the SecYEG translocon (5, 67), which can associate with either SecA (29, 61) or with the SRP receptor FtsY (13, 68) as peripheral receptor subunits for their respective client proteins. However, such classification does not seem to reflect the genuine complexity of bacterial protein transport. This is exemplified by the observation that SecA can interact with nontranslating ribosomes and that it can associate with its substrates even before translation is terminated (25, 38, 41), i.e., cotranslationally. SecA binds to ribosomes close to the ribosomal peptide tunnel exit (40). The tunnel exit is surrounded by four universally conserved proteins (uL22, uL23, uL24, and uL29) (69, 70), of which, in particular, uL23 is required for contacting ribosome-associated proteins like SRP (9, 71), trigger factor (TF) (26), SecY (72), and the nascent-chain-processing enzymes PDF and MAP (46, 73, 74). Our data now demonstrate that SecA also contacts uL23. This contact involves surface-exposed residues of uL23 but also the intratunnel loop, which is approximately 20 Å away from the tunnel exit. This is consistent with mutational studies, which demonstrate reduced SecA binding to ribosomes containing deletions of the intratunnel loop or replacements of the surface-exposed acidic patch ^51^FEVEVE^56^ in uL23 (40). So far, attempts to determine the ribosome-binding site within SecA have led to conflicting results. Based on mutational analyses, two lysine residues within the HLD of SecA (K^625^ and K^633^) were suggested to be required for ribosome binding (40). On the other hand, cryo-EM reconstructions of SecA bound to 70S ribosomes at low resolution identified the N terminus of SecA as a ribosome-binding site (50). These conflicting observations are now consolidated by our data, which show that both the N terminus of SecA and parts of the HLD, which includes residues K^625^ and K^633^, can penetrate the ribosomal polypeptide exit tunnel. The close proximity between the N terminus and the HLD (75) and a possible reorientation of the N terminus when parts of the HLD are missing could explain why deleting (Fig. 2) or mutating the HLD (40) interferes with SecA binding to the ribosome. The structure of the extended helix of the E. coli HLD likely requires rearrangements before it can insert into the ribosomal tunnel. Intriguingly, the HLD of Bacillus subtilis SecA forms a hairpin-like conformation (76) that could facilitate the HLD interaction with the ribosomal tunnel. Our data do not reveal whether the N terminus and parts of the HLD can insert simultaneously into the ribosomal tunnel. The terminal vestibule of the ribosomal tunnel is about 20 Å wide and was shown to accommodate folded or partially folded domains (51, 52); therefore, it would be wide enough to simultaneously harbor both the N terminus and parts of the HLD. However, this needs to be further analyzed.

The contribution of the N terminus to ribosome binding is of physiological relevance because the N terminus of SecA penetrates into the lipid bilayer when SecA associates with SecYEG (53), and it is directly involved in SecY binding (54, 55). The N terminus has also been linked to SecA dimerization (77, 78), to the SecB-dependent dissociation of the SecA dimer (65, 79), and to the topology inversion of SecG (80). This would indicate that the N terminus of SecA would be available only for ribosome interaction when SecA is in its soluble, monomeric state. This could explain why it has been difficult to model a SecA dimer on the ribosome (50) and is in line with our observation that the presence of membranes prevents cross-links between SecA and the ribosomal tunnel interior. The high affinity of SecA for anionic phospholipids and SecYEG with the consequence that the majority of SecA is bound to the membrane *in vivo* also explains why we were unable to detect the SecA-ribosome cross-links *in vivo*. However, it is important to emphasize that the *in vivo* interaction between SecA and translating ribosomes has been observed by cross-linking when SecA was overexpressed and by two-dimensional (2D) gel analyses (41).

The SecA-ribosome interaction is remarkably similar to the uL23-SRP interaction, where the SRP protein subunit Ffh inserts via its C terminus into the tunnel (10, 49). When a nascent protein chain approaches the intratunnel loop, SRP maintains contact to the surface-exposed residues of uL23, but the C terminus retracts into the proximal part of the tunnel (10). This reorientation probably primes the C-terminal M domain of SRP for efficient interaction with the emerging signal sequence (81–83). Displacement of SRP from the tunnel interior is observed with both SRP and SecA substrates (10), indicating that SRP decodes the sequence information only after the signal sequence has emerged from the ribosomal tunnel. The very same binding pattern is also observed for SecA; SecA loses contact with the intratunnel loop of uL23 in the presence of a nascent chain but maintains contact with the surface-exposed area of uL23. As observed for SRP, the retraction of SecA from the ribosomal tunnel is observed for canonical SecA substrates like OmpA, but also for SRP substrates, like LepB. The similar binding mode to the ribosome is in line with our data showing that SecA and SRP compete for ribosome binding and with the steric hindrance that is observed when SecA-ribosome and SRP-ribosome complexes are superimposed (50). Protrusion into the ribosomal peptide tunnel has so far only been observed for SecY (49), SRP (10), and for SecA (this study) but not for trigger factor, PDF, or MAP (10). This indicates that the intratunnel loop is particularly important for protein targeting and transport processes but not for nascent chain chaperoning or processing steps, which can occur after or in parallel with initiating targeting (46, 74, 84). By entering the ribosomal tunnel, SRP or SecA can scan the ribosomal tunnel for an emerging nascent chain and form stable complexes with their substrates once the RNC reaches a length of approximately 40 to 45 amino acids, in the case of SRP (10), or of approximately 100 amino acids in the case of SecA (41). The scanning mode of SRP is kinetically controlled by high dissociation rates in the absence of a canonical substrate (85). Whether this also applies for SecA needs to be further analyzed.

The simultaneous operation of co- and posttranslational targeting pathways to SecYEG has been rationalized by the limited number of SecYEG channels in the bacterial membrane. These channels are to a large extent occupied by translating ribosomes (86), and a posttranslational translocation by SecA would enable cells to rapidly translocate secretory proteins whenever a SecYEG channel is available. A general cotranslational transport mode by SecA therefore seems to be incompatible with the number of SecYEG channels. The preferential membrane binding of SecA and the inability of membrane-bound SecA to interact with ribosomes suggest that *in vivo*, only a smaller fraction of SecA is involved in cotranslational substrate recognition. This would also explain why we were unable to capture the SecA-ribosome contact in living cells by cross-linking. The efficiency of pBpa cross-linking is in the range of 10% (79), and the failure to detect cross-links *in vivo* could simply reflect a low abundance of SecA-ribosome complexes. However, even a small fraction of ribosome-bound SecA would reduce the need for secretion-specific chaperones, like SecB, and provide an explanation for the observation that only a few secretory proteins show reduced translocation when SecB is deleted (66, 87). In addition, cotranslational substrate recognition by SecA might be important for particular substrates, like the type II membrane protein RodZ, which is cotranslationally targeted and inserted by SecA (42, 43), or other single-spanning membrane proteins for which a targeting role of SecA has been suggested (44). Finally, because SecA, the SRP receptor FtsY, and ribosomes use overlapping binding sites on the SecYEG translocon (68), SecA is constantly displaced from the SecYEG translocon. The ability of the soluble SecA to interact with translating ribosomes could further increase the efficiency of protein translocation because substrate recognition by SecA would occur also independently of an available SecYEG translocon.

## MATERIALS AND METHODS

### Strains and plasmids.

Plasmids were propagated in E. coli DH5α (88). Proteins were expressed in E. coli strain BL21, BL21(DE3), or C43(DE3) (Novagen). E. coli MC4100 Δ*rplW*::*kan* (26), lacking the chromosomal uL23 gene (*rplW*) and supplemented with pCDFduet-L23 (11), was used for ribosome extraction. SecA-pBpa versions were expressed in the BL21(DE3) strain that had been previously transformed with pEVOL-aaRS (48) plasmid that encodes engineered tRNA and tRNA synthetase for pBpa incorporation at the TAG codon.

*rplW* pBpa and cysteine replacements and the deletion of 18 residues of the loop of uL23 were constructed with PCR using Phusion high-fidelity PCR kit (NEB, Ipswich, MA). The oligonucleotides that were used are listed in Table S1 in the supplemental material. The generation of pCDF-L23 (11) containing the TAG stop codon at different positions of *rplW* and the mutagenesis of pCDF-L23 G71Cys and pCDF-L23Δ18 variants was described by Denks et al. (10). pET19b-His_10_-SecA (89) served as the template for *secA* manipulations that were performed with PCR using the Phusion high-fidelity PCR kit (PCR oligonucleotides F/R1 to -9 in Table S1) or *Pfu*Ultra II high-sensitivity (HS) DNA polymerase (Agilent Technologies, Santa Clara, CA) (oligonucleotides F/R11 to -21). To control for a possible bias from the N-terminal His_10_ tag on protein interaction experiments, an HA tag was introduced instead with oligonucleotides F1 and R1 to obtain pET19b-HA-SecA. To construct C-terminally His-tagged SecA, *secA* was amplified with oligonucleotides F/R2 and -3; the pET19b vector that already encoded His_5_ before the stop codon was amplified with oligonucleotides F4 and R4, and the two PCR products were combined with Gibson Assembly (90).

10.1128/mBio.01317-19.1 TABLE S1 List of oligonucleotides used in this study. Listed are the functions of the oligonucleotide-dependent changes in *rplW*(uL23) and *secA*, the modified codons, the names of the oligonucleotides, and the nucleotide sequences. For details on the PCR-induced changes, see Materials and Methods. Download Table S1, DOCX file, 0.1 MB. Copyright © 2019 Knüpffer et al. 2019 Knüpffer et al. This content is distributed under the terms of the Creative Commons Attribution 4.0 International license.

SecA Δ611–621 and Δ884–901 mutants were also constructed with Gibson Assembly using oligonucleotides F/R5 to -9. The rest of the SecA deletion mutants were obtained with inverse-PCR using oligonucleotides F/R10 to -13. SecA pBpa versions (TAG replacements in s*ecA*) were constructed with oligonucleotides F/R14 and -15. For the cysteine mutants of SecA, the three endogenous cysteines (positions 885, 887, and 896) of SecA-His were replaced with serines using oligonucleotides F/R16 and -17. The fourth cysteine in position 98 was kept as an internal control for the specificity of the cross-linking. The resulting construct [SecA_His_(C_885_S/C_887_S/C_896_S)] was used as the template for site-specific cysteine replacements that were performed with oligonucleotides F/R18 to -21. All oligonucleotides used for PCR are shown in Table S1.

### Purification of proteins, ribosomes, RNCs, and INV.

SecA_His_ and their mutant versions were grown in LB medium. Isopropyl-β-d-thiogalactopyranoside (IPTG; 1 mM; Roth, Karlsruhe, Germany) was used to induce the cells at an *A*_600_ of 0.7 to 0.8. After 3 h of incubation, the cells were harvested, washed, and homogenized with the Emulsiflex C3 homogenizer (Avestin, Ottawa, Canada). Cell debris was removed at 30,000 × *g* for 20 to 30 min, and the cleared lysate was loaded on equilibrated Talon beads for 1 h. Talon-bound SecA was washed 4 times with wash buffer at pH 7.6 (50 mM HEPES-KOH, 1 M ammonium acetate, 10 mM magnesium acetate, 7 mM β-mercaptoethanol, 10% glycerol, 5 mM imidazole). The same buffer supplemented with 200 mM imidazole was used for protein elution. SecA was rebuffered using PD-10 desalting columns (GE Healthcare Life Sciences, Chalfont St. Giles, England) into CTF buffer (50 mM triethanolamine-acetate, 50 mM potassium acetate, 5 mM magnesium acetate) at pH 8.0.

N-terminally hemagglutinin-tagged SecA (HA-SecA) was expressed, harvested, and lyzed as described for SecA_His_, with the difference that buffer HA1 containing 20 mM Tris-HCl, 0.1 M NaCl, and 0.1 mM EDTA at pH 7.5 was used. After clarifying the cell lysate at 30,000 × *g* for 30 min, the supernatant was incubated with anti-HA-agarose (Thermo Fisher Scientific, Waltham, MA) for 1 h and washed thereafter 3 times with buffer HA1 containing 0.05% Tween 20 (Sigma-Aldrich, St. Louis, MO). The protein was eluted from the anti-HA-agarose with 1 mg/ml influenza HA peptide (Sigma-Aldrich) in buffer HA1. SRP was purified as described previously (10).

High-salt-washed wild-type 70S ribosomes were purified from strain MC4100. Ribosomes bearing pBpa at uL23 were purified from MC4100 Δ*rplW*::*kan* equipped with pCDF-L23(pBpa) and pSup-BpaRS-6TRN (47) for pBpa incorporation. uL23(Δ18-loop) ribosomes and uL23(G71Cys) ribosomes were purified from MC4100 Δ*rplW*::*kan.* The cells were propagated in medium consisting of 1% (g/wt) yeast extract, 1% (g/wt) tryptone-peptone, 41 mM KH_2_PO_4_, 166 mM K_2_HPO_4_, and 1% (g/wt) glucose (all components from Roth). Except for wild-type (wt) ribosomes, medium was supplemented with 50 μg/ml streptomycin (Sigma-Aldrich) and 0.5 mM IPTG (Roth). For pBpa incorporation, 35 μg/ml chloramphenicol (Sigma-Aldrich) and 0.5 mM pBpa (Bachem, Bubendorf, Switzerland) were added to the medium. When the cell density reached an *A*_600_ of 1.6 to 1.8, the growth was stopped on ice, and the cells were harvested, washed, and homogenized with the Emulsiflex C3 homogenizer. The lysate was cleared at 30,000 × *g* for 30 min and the crude ribosomes collected at 184,000 × *g* for 2.5 h. Ribosomes were dissolved in high-salt buffer (50 mM triethanolamine acetate, 1 M potassium acetate, 15 mM magnesium acetate, 1 mM dithiothreitol [DTT] [pH 7.5]) and purified through a 1.44 M sucrose cushion at 344,000 × *g* for 1 h. The 70S ribosomes were isolated through a 0.29 to 1.15 M sucrose gradient and centrifuged with a TH-641 swinging bucket rotor (Thermo Fisher Scientific) at 29,000 rpm for 17 h, concentrated at 344,000 × *g* for 1 h, and resuspended in CTF buffer at pH 7.5 with 1 mM DTT (Roth).

For *in vivo* production of RNCs, MC4100 Δ*rplW*::*kan* with pCDF-L23(pBpa) and pSup-BpaRS-6TRN expressing LepB or OmpA nascent chains from the pRha construct (10) based on the pRha-109 vector (91) (a gift from David Vikström, Stockholm University) was used. The cells were grown at 37°C in LB medium supplemented with 10 μg/ml tetracycline (Sigma-Aldrich), 50 μg/ml streptomycin, and 0.5 mM IPTG. The expression of nascent chains was induced at an *A*_600_ of 1.0 with 0.1% rhamnose (Sigma-Aldrich) for 1 to 2 h. An FtsQ, LepB, and OmpA RNC purification procedure followed a previously described protocol (10). SecYEG-overexpressing INVs were generated *in vivo* from the BL21 strain containing the plasmid pTrc99a-SecY_His_EG-YidC (16). INV purification was performed as described by Koch et al. (5).

### *In vitro* site-specific cross-linking.

For pBpa cross-linking, 500 nM E. coli 70S ribosomes or RNCs were combined with equimolar purified SecA and incubated 10 min at 30°C in CTF buffer with 1 mM DTT (pH 7.5). Cross-links were induced by UV exposure for 20 min on ice in a Biolink 365-nm cross-linking chamber (Vilber-Lourmat, Eberhardzell, Germany), and the control sample was kept in the dark. After trichloroacetic acid (TCA) precipitation, the samples were separated by SDS-PAGE and analyzed by Western blotting.

For the competition experiments between SecA and SRP, SecA (2.5 μM) and Ffh (0.5, 2.5, 12.5 μM) were premixed in buffer C (25 mM HEPES [pH 7.5], 70 mM ammonium acetate, 30 mM potassium acetate, 7 mM magnesium acetate, 10% glycerol) and incubated for 10 min at 30°C with 0.5 μM 70S ribosomes bearing pBpa in uL23. This was followed by UV cross-linking on ice for 20 min. The samples were TCA precipitated, separated by SDS-PAGE, and immunoblotted.

For cysteine cross-linking, 500 nM 70S ribosomes bearing a cysteine residue at position 71 of uL23 were incubated in CTF buffer without DTT for 10 min at 30°C with equimolar concentrations of purified SecA lacking the cysteine residues at the C terminus, as described above. Cross-linking was performed for both wild-type SecA and SecA variants that contained a cysteine residue at position 2, 3, or 5. For inducing disulfide bonds, 100 μM sodium tetrathionate (NaTT; Sigma-Aldrich) was added. In the control reactions, 0.5 mM TCEP was used instead. All samples were incubated for 5 min at 25°C and subsequently precipitated with 5% TCA. Thereafter, the samples were separated on SDS-PAGE and detected by Western blotting.

For cysteine cross-linking in the presence of INVs, SecA variants (1 μM) were first incubated with SecYEG-YidC-overexpressing INVs (2 μM) for 10 min at room temperature in CTF buffer without DTT containing sucrose (50 mM triethanolamine-acetate, 50 mM potassium acetate, 5 mM magnesium acetate, 250 mM sucrose). The material was centrifuged at 110,000 × *g* for 30 min to remove unbound SecA. After the addition of 70S ribosomes containing a cysteine residue at position 71 of uL23 and incubation for 10 min at 30°C, cysteine cross-linking was induced with 200 μM of NaTT. Control reactions were treated with TCEP. Incubation for 5 min at 25°C and TCA precipitation followed for all samples. The material was analyzed by SDS-PAGE and Western blotting.

### *In vivo* site-specific cross-linking.

MC4100 ΔrplW::kan pCDF-L23/pSup-BpaRS-6TRN strains containing either wild-type uL23 or uL23 variants with pBpa at position 71 or 52 were grown in S130 medium (1% [g/wt] yeast extract, 1% [g/wt] tryptone-peptone, 41 mM KH_2_PO_4_, 166 mM K_2_HPO_4_, and 1% [g/wt] glucose), supplemented with 50 μg/ml streptomycin, 0.5 mM IPTG, and 0.5 mM pBpa for pBpa incorporation at 37°C until the exponential-growth phase was reached (optical density at 600 nm [OD_600_], 1.0). The cells were harvested at room temperature, and the cell pellets were resuspended in phosphate-buffered saline (PBS) buffer (137 mM NaCl, 2.7 mM KCl, 10 mM Na_2_HPO_4_, and 1.76 mM KH_2_PO_4_) at a 1:2 (pellet in grams/buffer in milliliters) ratio. The resuspended material was distributed into 6-well plates and kept on ice. Half of the material was exposed to UV light, while the other part was kept in the dark. Subsequently, the cells were collected by centrifugation at 5,000 rpm for 10 min in a tabletop centrifuge, resuspended in CTF buffer, and homogenized with an Emulsiflex C3 homogenizer. The cell debris was removed by centrifugation at 30,000 × *g* for 30 min, followed by further centrifugation at 184,000 × *g* for 2.5 h to collect membranes and crude ribosomes.

To analyze the cross-links in the cytosolic ribosome fraction, the pellets were dissolved in high-salt buffer, and the ribosomes were separated through a 1.44 M sucrose cushion. After resuspension in CTF buffer, 6 μM of the ribosome material was loaded on SDS-PAGE and analyzed by immunoblotting. For examination of the membrane fraction, the pellets were instead resuspended in buffer A (50 mM triethanolamine-acetate, 250 mM sucrose, 1 mM EDTA, 1 mM DTT, protease inhibitors), and inner membrane vesicles were purified through a sucrose step gradient (0.77 to 1.44 to 2.02 M), as previously described (5). Three hundred micrograms of the INV material was analyzed by SDS-PAGE and Western blotting.

### Antibodies.

Polyclonal antibodies against SecA and SRP were raised in rabbits (5, 92). Antibodies against E. coli uL23 raised in sheep were a gift from Richard Brimacombe (Max-Planck-Institut für Molekulare Genetik, Berlin, Germany). Monoclonal anti-His antibodies were from Roche Applied Science and HA epitope tag antibodies from Thermo Fisher Scientific.
