# Supplementary material for: Molecular Mimicry of SecA and Signal Recognition Particle Binding to the Bacterial Ribosome
Source: mBio. 2019 Aug 13;10(4):e01317-19. doi: 10.1128/mBio.01317-19 (PMC6692507; doi:10.1128/mBio.01317-19)
Supplement: TABLE S1 [file mBio.01317-19-st001.docx]

Supplementary material to

**Molecular mimicry of SecA and SRP binding to the bacterial ribosome**

**Supplementary Table S1**

List of oligonucleotides used in this study. Listed are the functions of the oligonucleotide-dependent changes in *rplW*(uL23) and *secA*, the modified codons, the names of the oligonucleotides and the nucleotide sequence. For details on the PCR-induced changes see material and methods.

| **Function** | **Mutation** | **Name** | **Sequence** |
| --- | --- | --- | --- |
| Incorporation of pBpa  into uL23 | E18TAG | E18F | caccgcacgtttcttagaaagcgtctactgc |
|  |  | E18R | cacgcagcaccttcagcagacgttcttcacg |
|  | E42TAG | E42F | tgcagaaactgtttgaagtcgaagtcgaagtc |
|  |  | E42R | cagcagctttgatctatgctttggtcgc |
|  | E52TAG | E52F | cagaaactgttttaggtcgaagtcgaag |
|  |  | E52R | cacagcagctttgatttctgctttggtc |
|  | G71TAG | G71F | gtcgtagcgactggaaaaaagc |
|  |  | G71R | gaccgatacgctgctagtgacgtttaac |
| Incorporation of cysteine to  uL23 | G71C | G71F | gtcgtagcgactggaaaaaagc |
|  |  | G71C R | gaccgatacgctggcagtgacgtttaac |
| Loop deletion of uL23 | Δ62-79 | -11F | cagggtgttaacgacttcgacttcgacttcaaacag |
|  |  | -11R | tggaaaaaagcttacgtcaccctgaaagaaggccag |
| Incorporation of N-terminal HA-tag  to SecA | HA-SecA | F1 | ttccagattacgctagcagcggccatatcgac |
|  |  | R1 | catcgtatgggtacatggtatatctccttcttaaag |
| Moving His-Tag to C-terminus of SecA via Gibson assembly |  | F2 | ggagatatacccacatgctaatcaaattgttaac |
|  |  | R2 | atggtgatgttgcaggcggccatga |
|  |  | F3 | taagaaggagatatacccacatgct |
|  |  | R3 | gcttaatggtgatggtgatgttgc |
|  |  | F4 | catcaccatcaccattaa |
|  |  | R4 | gtgggtatatctccttctta |
| Constructing SecA  deletions | ∆611-621 | F5 | aagctaactgttgaagtaaagc |
|  |  | R5 | atgcttgtcgtcgtcgtc |
|  |  | F6 | tcgacgacgacgacaagcatatgctaatcaaattgttaactaaagttttc |
|  |  | R6 | tagtcacccacagtttacgcatcatgcc |
|  |  | F7 | gcgtaaactgtgggtgactaaagcgattg |
|  |  | R7 | tttacttcaacagttagcttttattgcaggcggccatg |
|  | ∆884-901 | F8=F5 | aagctaactgttgaagtaaagc |
|  |  | R8=R5 | atgcttgtcgtcgtcgtc |
|  |  | F9 | tcgacgacgacgacaagcatatgctaatcaaattgttaactaaagttttc |
|  |  | R9 | tttacttcaacagttagcttttaatcgttacgtcctactttg |
|  | ΔPBD  (233-365) | F10 | gctggtcagaacgaaaaccaaacgct |
|  |  | R10 | accttccaggctgtcttctgccg |
|  | Δ604-636 | F11 | cgtaacttcgacattcgtaagcaactgc |
|  |  | R11 | tactcggtcggaagcaaaaatacgcatc |
|  | ΔN9 | F12 | ggtagtcgtaacgatcgc |
|  |  | R12 | catgtgggtatatctccttcttaaagttaaac |
|  | ΔN18 | F13 | cggatgcgcaaagtgatcaacat |
|  |  | R13=R12 | catgtgggtatatctccttcttaaagttaaac |
| Incorporation of pBpa  into SecA | 615pBpa | F14 | ctgggtatgaagccataggaagccattgaac |
|  |  | R14 | tttacgcatcatgccggatactcggtc |
|  | 5pBpa | F15 | tagttaactaaagttttcggtagtcgtaacg |
|  |  | R15 | tttgattagcatgtgggtatatctccttc |
| Deleting the C-terminus of SecA with 3 Cysteines |  | F16 | catcaccatcaccattaagcttatcgatga |
|  |  | R16 | atcgttacgtcctactttgcgctcacc |
| Restoring the Cys-free C-terminus (Ser885,887,896) |  | F17 | tataagcagtctcatggccgcctgcaacatcaccatcaccattaag |
|  |  | R17 | ttttttaccagaacctgacggtgaaggatcgttacgtcctacttt |
| Incorporation of  cysteine residues  into SecA | 2Cys | F18 | atcaaattgttaactaaagttttcggtagtcg |
|  |  | R18 | acacatgtgggtatatctccttcttaaagttaaac |
|  | 3Cys | F19 | gatatacccacatgctatgcaaattgttaacta |
|  |  | R19 | tccttcttaaagttaaacaaaattatttctagaggg |
|  | 5Cys | F20 | tgtttaactaaagttttcggtagtcgtaacg |
|  |  | R20=R15 | tttgattagcatgtgggtatatctccttc |
|  | 625Cys | F21 | tgtgcgattgccaacgcccagcg |
|  |  | R21 | agtcacccacgggtgttcaatggcttcg |
